# RXR*α* Blocks Nerve Regeneration after Spinal Cord Injury by Targeting p66shc

**DOI:** 10.1155/2021/8253742

**Published:** 2021-02-10

**Authors:** Pei Yu, Kai Yang, Min Jiang

**Affiliations:** ^1^Department of Orthopedics, Ruijin Hospital Affiliated to Shanghai Jiao Tong University School of Medicine, 97 Ruijin 2nd Road, Shanghai 200025, China; ^2^Shanghai Key Laboratory for Prevention and Treatment of Bone and Joint Diseases, Shanghai Institute of Traumatology and Orthopaedics, Ruijin Hospital, Shanghai Jiao Tong University School of Medicine, 197 Ruijin 2nd Road, Shanghai 200025, China

## Abstract

Nerve regeneration after spinal cord injury is regulated by many factors. Studies have found that the expression of retinoid X receptor *α* (RXR*α*) does not change significantly after spinal cord injury but that the distribution of RXR*α* in cells changes significantly. In undamaged tissues, RXR*α* is distributed in motor neurons and the cytoplasm of glial cells. RXR*α* migrates to the nucleus of surviving neurons after injury, indicating that RXR*α* is involved in the regulation of gene expression after spinal cord injury. p66shc is an important protein that regulates cell senescence and oxidative stress. It can induce the apoptosis and necrosis of many cell types, promoting body aging. The absence of p66shc enhances the resistance of cells to reactive oxygen species (ROS) and thus prolongs life. It has been found that p66shc deletion can promote hippocampal neurogenesis and play a neuroprotective role in mice with multiple sclerosis. To verify the function of RXR*α* after spinal cord injury, we established a rat T9 spinal cord transection model. After RXR*α* agonist or antagonist administration, we found that RXR*α* agonists inhibited nerve regeneration after spinal cord injury, while RXR*α* antagonists promoted the regeneration of injured neurites and the recovery of motor function in rats. The results showed that RXR*α* played an impeding role in repair after spinal cord injury. Immunofluorescence staining showed that p66shc expression was upregulated in neurons after spinal cord injury (*in vivo* and *in vitro*) and colocalized with RXR*α*. RXR*α* overexpression in cultured neurons promoted the expression of p66shc, while RXR*α* interference inhibited the expression of p66shc. Using a luciferase assay, we found that RXR*α* could bind to the promoter region of p66shc and regulate the expression of p66shc, thereby regulating nerve regeneration after spinal cord injury. The above results showed that RXR*α* inhibited nerve regeneration after spinal cord injury by promoting p66shc expression, and interference with RXR*α* or p66shc promoted functional recovery after spinal cord injury.

## 1. Introduction

Nerve regeneration after spinal cord injury is a key determinant that hinders functional recovery in injured bodies. Repair after spinal cord injury is affected by many factors, including the ability of neurons to repair themselves, the proliferation of astrocytes, the inflammatory response induced by microglia and the ability to remove apoptotic necrotic tissues, and the remyelination of oligodendrocytes. Of these, the repair ability of neurons themselves after injury is a key determinant of neurite regeneration [1]. Cholesterol is an important component in the formation of nerve cell membranes and myelin sheaths. The free cholesterol content in the brain accounts for 1/4 of the total cholesterol content in the whole body. Cholesterol in the brain is typically synthesized by the brain itself because cholesterol cannot pass through the blood-brain barrier. The synthesis, storage, transport, and exclusion of cholesterol maintain a dynamic balance, playing a crucial role in the formation and maintenance of neuroplasticity and the execution of various functions [2]. Studies have found that retinoic acid receptors (RARs) and retinoic X receptors (RXRs) form heterodimers as ligand-activated transcription factors to promote axon regeneration and affect glial differentiation and the regulation of inflammation [3, 4]. The RXR*α*-mediated transmembrane cholesterol transport system plays an important role in maintaining the balance of intracellular and extracellular cholesterol. As a ligand transcription factor, RXR*α* plays an important role in regulating cell growth, differentiation, death, and metabolism. Studies have found that the downregulation of RXR*α* expression can promote the regeneration of the caudal spinal cord of adult salamanders [5]. RXR*α* is expressed in the cytoplasm of neurons, astrocytes, and some oligodendrocytes before spinal cord injury; after spinal cord injury, RXR*α* is expressed in the nucleus of neurons, astrocytes, and activated microglia [3]. After entering the nucleus, RXR*α* can interact with RAR*β* and other molecules to bind to the promoter region of downstream regulatory genes and participate in promoting or inhibiting the expression of downstream signaling proteins [6]. p66shc protein, which is encoded by the protooncogene shcA, is involved in the regulation of reactive oxygen species (ROS) levels and age-related organ dysfunction. Knockout of the p66shc gene can prolong the lifespan of mice. p66Shc is highly expressed in neural stem cells and neural precursor cells, but its expression is significantly reduced in adult individuals [7]. Studies have shown that the expression level of brain-derived neurotrophic factor (BDNF) at the base of the hippocampus in p66shc^−/−^ mice is significantly increased, thereby improving the cognitive ability and anti-inflammatory ability of mice [8]. Studies have found that p66shc plays a role in the regulation of redox and energy metabolism in neurons [9]. p66shc can also be used as a regulator of neuronal differentiation and proliferation during neuron development [10]. Therefore, we speculate that p66shc plays an important role in repair after spinal cord injury. Our study found that RXR*α* inhibited neurite regeneration and functional recovery after spinal cord injury in rats. The expression of p66shc was upregulated after spinal cord injury, and p66shc colocalized with RXR*α*. We also demonstrated that RXR*α* inhibited neurite regeneration after spinal cord injury by downregulating p66shc expression. Currently, there are few studies on the function of RXR*α* and p66shc after spinal cord injury. Therefore, in this study, we focused on the role of RXR*α* in the regeneration of neurites after spinal cord injury via regulating the expression of p66shc.

## 2. Material and Methods

### 2.1. Rat Spinal Cord Injury Model

Animal experiments were approved by the ethics review committee of Ruijin Hospital affiliated with Shanghai Jiao Tong University, China. A total of 360 Sprague-Dawley (SD) rats (8-week-old, male) were purchased from Shanghai Laboratory Animal Center (SLAC), China. Animals were housed in a ventilated room at constant temperature (24°C), in 50-60% humidity, and under a 12-hour light/dark cycle, with standard rat diet and free access to water. After 1 week of feeding, a dorsal hemisection was performed at the T9 segment of the spinal cord. Rats that only underwent laminectomy and hemisection were used as the sham control group. The bladder was manually emptied every 8 hours after injury. Three hundred sixty rats were randomly divided into 4 groups: sham control group (*n* = 90), injury group (*n* = 90), RXR*α* agonist group (*n* = 90), and RXR*α* antagonist group (*n* = 90). Five rats in each group were used for tissue sectioning, 5 rats were used for RNA extraction, and 5 rats were used for protein extraction. Each group of rats was evaluated for motor function at 3 days, 1 week, 2 weeks, 3 weeks, 4 weeks, and 5 weeks after spinal cord injury, and tissues were collected to be used in the above experiments after motor function evaluation. The RXR*α* agonist CD3254 (MCE, USA) was administered at 100 mg/kg/day by gavage, and the RXR*α* antagonist PA452 (MCE, USA) was administered at 1 mg/kg/day by gavage. Drugs were given starting the first day after injury until tissue collection.

### 2.2. Primary Neuron Culture and *In Vitro* Injury Model

Rat spinal cord tissue from 18-day-old embryos was collected for primary spinal cord neuron culture. Tissues were removed and digested with 0.05% trypsin-ethylene diamine tetraacetic acid (EDTA) (Thermo Scientific, USA) at 37°C for 15 min. After the cells were pipetted into a single cell suspension, Dulbecco's modified Eagle's medium (DMEM)+10% fetal bovine serum (FBS) (Thermo Scientific, USA) was added and mixed, and then, the cells were seeded in 6-well plates. After 4 hours, the culture medium was replaced with Neurobasal+1% N_2_+2% B27 (Thermo Scientific, USA) culture medium for 3-10 days. Primary spinal neurons cultured *in vitro* were used for the scratch assay after 3 days. A cell scraper or 20 *μ*l pipette tip was used to scratch the cell monolayer. *In vitro* culture was continued for 7-10 days to observe the regeneration of neurites after *in vitro* neuronal injury. The final concentrations of the RXR*α* agonist CD3254 and RXR*α* antagonist PA452 in the *in vitro* culture system were 50 *μ*g/l and 0.1 *μ*M, respectively.

#### 2.2.1. Primary Spinal Cord Slice Culture

SD rat embryonic (E) 18-day-old rat spinal cord tissue (thoracolumbar spinal cord segment) was collected for spinal cord slice culture. After cutting the spinal cord tissue into thin slices with microscissors, tissues were placed on a coverslip precoated with Matrigel (R&D, USA). The coverslips were placed into a 24-well plate, 200 *μ*l of culture medium (DF12+10% FBS) was then added, and the plate was placed in an incubator to culture for 4 hours; subsequently, 300 *μ*l of medium (DF12+10% FBS) was added, and the cells were cultured for another 7 days, with the medium being changed every other day. After 7 days of culture, the growth of neurites was observed. If the growth of the neurites was good, the neurites were removed along the spinal cord slices with a blade under a microscope, and the *in vitro* culture was continued for 7-10 days to observe the regeneration of neurites after *in vitro* neuronal injury. The measurement of neurites was performed using 3 slices per group, and 10 fields of view (200x magnification) were observed under a microscope (Olympus, Japan) for each slice. The lengths of neurites in all images were measured and averaged. Comparisons between groups were performed. In the spinal cord slices, the lengths of all neurites were measured and compared between groups.

### 2.3. Immunohistochemical Staining and Immunofluorescence Staining

Rats were anesthetized with 2% sodium pentobarbital (Sinopharm Group Co., Ltd., China), perfused with 100 ml of normal saline (Sinopharm Group Co., Ltd., China), and fixed with 200 ml of 4% paraformaldehyde (Beyotime, China). Spinal cord tissue was removed and placed in paraformaldehyde at 4°C overnight and then sequentially replaced with 20% sucrose (Sinopharm Group Co., Ltd., China) and 30% sucrose for dehydration; the samples were then used for frozen tissue sectioning. The thickness of each frozen section was 15-20 *μ*m. The tissue sections were placed on a metal sectioning rack in a beaker containing sodium citrate, an antigen retrieval solution (Beyotime, China). The antigen retrieval solution in the beaker covered the tissue sections. The solution was heated to 95°C for 15 min, followed by natural cooling to room temperature; then, the sections were washed with 0.01 M PBS (Beyotime, China) for 5 min, 3 times, and incubated with 3% H_2_O_2_ (Beyotime, China) at room temperature for 10 min to inactivate endogenous peroxidases. The samples were then washed with 0.01 M PBS for 5 min, 3 times, and incubated with blocking solution containing 5% normal goat serum (Beyotime, China) at room temperature for 30 min. Rat *β*-III-tubulin antibody (Abcam, USA) was diluted 1 : 1000 in primary antibody dilution solution (Beyotime, China), mixed well, added dropwise onto the surface of the tissue, and incubated at 37°C for 2 hours, followed by the dropwise addition of polymerized HRP-labeled anti-rabbit/rat IgG (Beyotime, China). The samples were incubated at 37°C for 30 min and washed with PBS for 2 min, 3 times; DAB was used for color development. Immunofluorescence staining was performed as follows. Tissue sections or coverslips were incubated with blocking solution containing 5% normal goat serum for 30 min at room temperature. Rat *β*-III-tubulin antibody was diluted 1 : 500, rat RXR*α* antibody was diluted 1 : 500 (Abcam, USA), and rabbit p66shc antibody was diluted 1 : 200 in primary antibody dilution solution, mixed well, and added dropwise to the surface of the tissue; the samples were incubated at 4°C overnight and then washed with 0.01 M PBS for 10 min, 3 times. Goat anti-mouse 488 secondary antibody and goat anti-rabbit 555 secondary antibody (Thermo Scientific, USA) were diluted 1 : 500 in secondary antibody dilution solution, mixed well, and added dropwise to the surface of tissues or cells; the samples were incubated at 37°C for 1 hour and then washed with 0.01 M PBS for 10 min, 3 times. DAPI (Thermo Scientific, USA) staining solution was added dropwise to the tissue sections. The slices were covered with coverslips, and the staining results were observed under an upright fluorescence microscope (Olympus, Japan).

### 2.4. Lentivirus Transfection and Luciferase Assay

Lentiviruses for the interference and overexpression of RXR*α* and p66shc were purchased from Shanghai Genechem Co., Ltd., China, with a titer of 1 × 10^8^ TU/ml. Neurobasal medium was used to prepare a suspension of primary neuronal cells at a density of 5 × 10^6^ cells/ml, and the cells were seeded in 6 cm Petri dishes. When the inoculation density reached more than 90%, 400 *μ*l of virus infection enhancement solution HitransG A and 100 *μ*l of lentivirus or negative control (neg-control), each with a titer of 1 × 10^8^ TU/ml, were added, and the cells were incubated at 37°C for 24 hours. The medium was changed every other day for continued culture. Approximately 72 hours after infection, the infection efficiency (number of GFP-positive cells) was observed. When the efficiency reached or exceeded 90%, infection was considered successful, and subsequent experiments were performed.

### 2.5. Evaluation of Motor Function after Spinal Cord Injury

The assessment of motor function after spinal cord injury was performed using the Basso Mouse Scale (BMS) motor score, the rotarod test, and footprint length. BMS motor scoring involved placing the rats in an open field (diameter: 100-200 cm), after which the rats were observed by 2 trained observers for 4 min; the score ranged from 0 to 9 points (0 points: complete hindlimb paralysis; 9 points: normal motor function). The results of this study were based on the results of hindlimb motor performance in an open field, including hindlimb joint movement, weight support, plantar stepping, coordination, paw position, and trunk and tail control. For the rotarod test, pretrained animals were placed on a carousel with an accelerating rotation speed of 3 to 15 rpm (revolutions per minute) for more than 2 min. Five tests were conducted each time. The latent period of the rats on the rotating rod was recorded according to the time when the rats fell in each experiment, and then, the times were averaged to obtain the final result for each experiment. Footprint length was measured using an unbiased step-scanning device. All rats walked on the electronic treadmill belt at a speed of 11 cm/s for 20 seconds. The digital data (footprint and body movement) were analyzed using TreadScan software (CleverSys Inc., Reston, VA). Each parameter was compared between the ipsilateral and contralateral sides and between the treatment group and the control group.

### 2.6. Western Blot

Tissue or cellular protein was extracted and subjected to BCA protein quantification to determine protein concentration. Sodium dodecyl sulfate-polyacrylamide gel electrophoresis (SDS-PAGE) was performed. Twenty micrograms of total protein was loaded onto each well for gel electrophoresis. The proteins were transferred to a PVDF membrane (Millipore, USA) and blocked at room temperature for 30 min. Primary antibodies, i.e., mouse polyclonal RXR*α* antibody (Abcam, USA), rabbit polyclonal shc antibody (Abcam, USA), and the corresponding secondary antibodies, i.e., goat anti-mouse HRP (CST, USA) and goat anti-rabbit HRP (CST, USA), were used to protein bands. Protein quantification was performed using IPP software. The differences between groups were compared.

### 2.7. Real-Time PCR

Samples were collected according to the conventional RNA extraction method. One milliliter of TRIzol (Invitrogen, USA) was added to cells and vascular tissues, and then, 200 *μ*l of chloroform (Sinopharm Group Co., Ltd., China) was added; the samples were mixed, allowed to stand for 10 min, and centrifuged at 12000 rpm for 15 min at 4°C. The upper aqueous phase was transferred to a new RNase-free EP tube. Five hundred microliters of isopropanol was added, and the samples were mixed well. After standing for 10 min, the samples were centrifuged at 12000 g for 10 min. The supernatant was discarded, and the RNA pellet was washed with 75% ethanol and dried. When the RNA pellet became colorless and transparent, 30 *μ*l of diethyl pyrocarbonate (DEPC) water was added to dissolve the RNA, which was then stored at -80°C. Quantitative PCR detection of genes was performed using a TB Green® Premix Ex Taq™ kit (Takara, Japan). A 20 *μ*l reaction system was used, with the following reaction conditions: predenaturation at 95°C for 30 seconds, followed by 40 cycles of 95°C for 5 seconds and 60°C for 30 seconds. The amplification curve and melting curve were confirmed after the reaction. The relative amount of the detected gene was calculated according to the formula 2-^*ΔΔ*CT^. Comparisons between groups were performed. The following were the primer sequences: p66shc: forward: 5′-GATTCAATTCCGGAGTTCTTA-3′, reverse: 5′-TTAGATCCATTCCCGAATTG-3′ and GADPH: forward: 5′-CTTGCTCAAGCTTAGTTCTAGG-3′, reverse: 5′-GAGTGCTCAGTGGTATTGC -3′.

### 2.8. Luciferase Assay

Primary cultured neurons were seeded in 6-well plates at a density of 2 × 10^5^ per well. Lentivirus containing either PDS131_psiCHECK-2 blank vector (6273 bp), PDS131_psiCHECK-2-p66shc wild-type, or PDS131_psiCHECK-2-p66shc mutant (mut, deletion mutation of “GGCTATTCGTA” in the promoter region of the wild-type p66shc gene) (Shanghai Genechem Co. Ltd., Shanghai, China) was added to neurons, with an inoculation density above 90%. After 72 hours of transfection, the luciferase activity in each group was detected using a dual-luciferase reporter gene detection system (Beyotime, China). First, the corresponding value of the fluorescence emitted by firefly luciferase- (FL-) induced substrate was detected using a microplate reader, and then, the fluorescence released by Renilla luciferase- (RL-) induced substrate was detected using a microplate reader. The measured FL to RL ratio for each sample was used as the relative luciferase activity of the reporter gene.

### 2.9. Statistical Analysis

All statistical results were analyzed using SPSS 17.0 (SPSS Inc., Chicago, USA). All quantitative indicators are expressed as the mean ± SD. The experimental data were analyzed using one-way analysis of variance and Tukey's post hoc test. *P* < 0.05 indicated significant differences between groups.

## 3. Results

### 3.1. RXR*α* Inhibited Neurite Regeneration after Spinal Cord Injury in Rats

**Figure 1:**
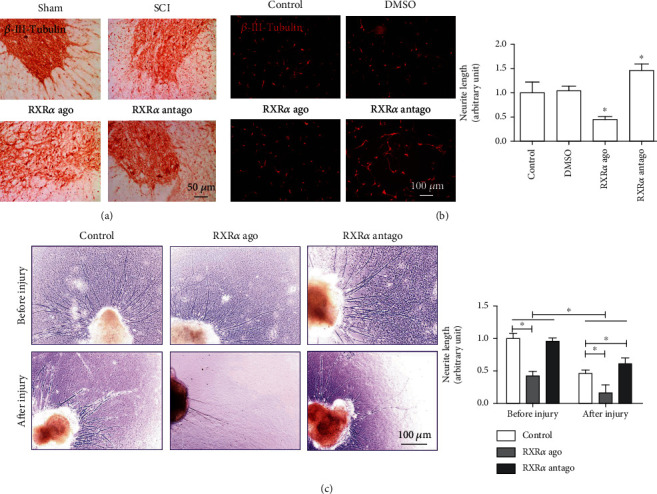
RXR*α* inhibited nerve regeneration after spinal cord injury. (a) *β*-III-Tubulin immunohistochemical staining showed that after spinal cord injury, the number of neuronal cells in the injured area was reduced, and the neurite continuity was interrupted. After RXR*α* agonist administration, the morphology of the tissue lacked integrity, and the neurons were in an apoptotic state; there were no continuous neurites. After RXR*α* antagonist administration, the number of neuronal cells increased, the cell morphology was good, and there was some neurite regeneration. (b) *β*-III-Tubulin immunofluorescence staining of primary spinal cord neuronal cells cultured *in vitro* showed that after scratch damage, the length of neurites in the RXR*α* agonist group was significantly shorter than that of neurites in the control group, while the length of neurites in the RXR*α* antagonist group was significantly longer than that of neurites in the control group. The results suggest that RXR*α* inhibits neurite regeneration after spinal cord injury. (c) Compared with that of neurites in the postinjury control group, the length of the regenerated neurites in the spinal cord slices cultured *in vitro* in the RXR*α* agonist group was significantly shortened after injury, while the length of the regenerated neurites in the RXR*α* antagonist group significantly increased. The length of postinjury neurites in each group was significantly shorter than that before injury. *N* = 5/group; ^∗^vs. control, *P* < 0.05, scale bar: 50 or 100 *μ*m.

First, we clarified the role of RXR*α* in neurite regeneration after spinal cord injury. The results showed that an RXR*α* agonist inhibited neuronal repair after spinal cord injury, while an RXR*α* antagonist promoted neuronal repair after spinal cord injury. Furthermore, the RXR*α* agonist caused a large number of neurons around the injury site to lose their normal morphological characteristics; that is, they were in an apoptotic state, making it difficult for neurite regeneration. The RXR*α* antagonist protected neurons after spinal cord injury and maintained their morphological characteristics; neurite regeneration was superior to that in the spinal cord injury group (Figure 1(a)). *In vitro* cultured neuronal injury experiments also showed that after neuronal injury, the administration of the RXR*α* agonist led to neurite growth suppression, while the administration of the RXR*α* antagonist promoted neurite growth, results that were significantly different from those in the control group (Figure 1(b)). We also used spinal cord slice culture to further clarify the role of RXR*α* in the regeneration of spinal cord neurites. When the neurites were cut along the periphery of the spinal cord slices and the RXR*α* agonist or RXR*α* antagonist was administered, it was found that in the RXR*α* agonist group, spinal cord slices only grew a small number of neurites, and their lengths were short; in the RXR*α* antagonist group, neurite regeneration was significantly promoted after injury. We also observed that the addition of the RXR*α* agonist to the spinal cord slices before injury also inhibited neurite growth (Figure 1(c)). The above results indicate that RXR*α* can inhibit the growth and regeneration of spinal cord neurites.

### 3.2. RXR*α* Inhibited Functional Recovery after Spinal Cord Injury in Rats

**Figure 2:**
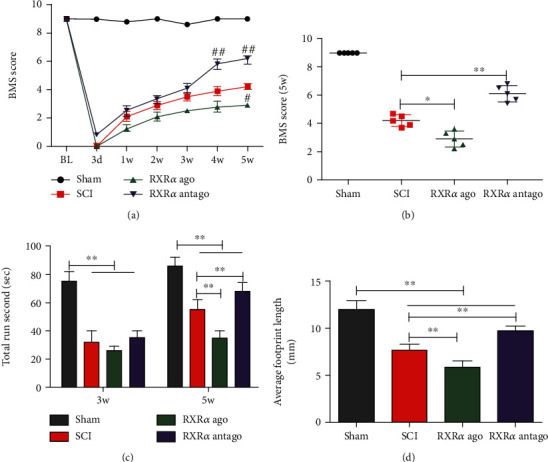
RXR*α* inhibited functional recovery after spinal cord injury in rats. (a) The BMS motor scores of rats in all groups were close to 0 at 3 days after injury. The motor scores gradually increased with the injury time. At 4 weeks after injury, rats that received RXR*α* antagonist had significantly higher motor scores than did rats in the spinal cord injury group, whereas the rats that received RXR*α* agonist had significantly lower motor scores than did rats in the spinal cord injury group 5 weeks after injury. (b) Five weeks after injury, the BMS motor scores of rats that received RXR*α* agonist were significantly lower than those of rats in the spinal cord injury group, and the BMS motor scores of rats that received RXR*α* antagonist were significantly higher than those of rats in the spinal cord injury group. (c) Rotarod test results showed that neither the RXR*α* agonist nor antagonist affected the rotarod test time for rats at 3 weeks after injury. The rotarod test time for rats given the RXR*α* agonist was significantly shorter than that for rats in the spinal cord injury group at 5 weeks after spinal cord injury. The rotarod test time for rats given RXR*α* antagonist was significantly longer than that for rats in the spinal cord injury group, indicating that RXR*α* hindered the recovery of motor function after spinal cord injury in rats. (d) Footprint length experiments showed that the distance between the 2 footprints for rats in the RXR*α* agonist group was significantly shorter than that for rats in the control group and the spinal cord injury group, while the distance between 2 footprints was significantly longer for the rats in the RXR*α* antagonist group than that for rats in the control group; however, recovery to the preinjury level was not attained. *N* = 5/group. ^∗^vs. control, *P* < 0.05; ^∗∗^vs. control, *P* < 0.01; ^#^vs. spinal cord injury (SCI) group, *P* < 0.05; ^##^vs. SCI group, *P* < 0.01.

Because RXR*α* inhibited the regeneration of spinal cord neurites, does RXR*α* affect the recovery of motor function in rats after spinal cord injury? We used the BMS motor score, rotarod test, and footprint length to observe the motor function of rats at 3 days, 1 week, 2 weeks, 3 weeks, 4 weeks, and 5 weeks after spinal cord injury. The results showed that the BMS motor scores of rats in all groups were close to 0 at 3 days after injury. The motor scores gradually increased with the injury time. At 4 weeks after injury, the motor scores of rats treated with the RXR*α* antagonist were significantly higher than those of rats in the spinal cord injury group. At 5 weeks after injury, the motor scores of the rats treated with RXR*α* agonist were significantly lower than those of rats in the spinal cord injury group (Figures 2(a) and 2(b)), indicating that inhibition of RXR*α* can promote functional recovery after spinal cord loss in rats. The results of the rotarod test showed that neither the RXR*α* agonist nor antagonist affected the rotarod test time for rats at 3 weeks after injury. Five weeks after spinal cord injury, the rotarod test time of rats treated with the RXR*α* agonist was significantly shorter than that of rats in the spinal cord injury group, while the rotarod test time of rats treated with the RXR*α* antagonist was significantly longer than that of rats in the spinal cord injury group, which also showed that RXR*α* blocked the recovery of motor function after spinal cord injury in rats (Figure 2(c)). Footprint length measurement experiments also showed that RXR*α* blocked the recovery of motor function after spinal cord injury in rats (Figure 2(d)). Based on the above research results, RXR*α* inhibits functional recovery after spinal cord injury in rats.

### 3.3. The Expression of p66shc Was Upregulated after Spinal Cord Injury and Colocalized with RXR*α*

**Figure 3:**
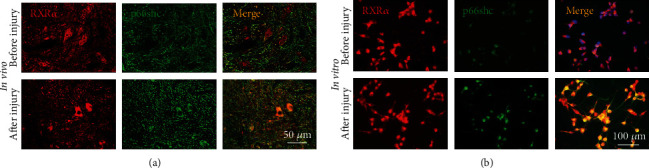
Expression of p66shc before and after spinal cord injury. Immunofluorescence staining showed that there was no significant difference in the expression of RXR*α* before and after spinal cord injury (rat spinal cord tissue (a) and primary neurons cultured *in vitro* (b)), while p66shc was expressed at low levels before spinal cord injury and the expression significantly increased after spinal cord injury. p66shc was colocalized with RXR*α*. *N* = 5/group, scale bar: 50 or 100 *μ*m.

As a ligand-activated transcription factor, RXR*α* regulates downstream signaling molecules and is involved in the inhibition of neurite regeneration after spinal cord injury. We used immunofluorescence staining to find that p66shc, a protein associated with cell senescence and oxidative stress, was expressed at low levels before spinal cord injury; p66shc expression significantly increased after spinal cord injury, and p66shc colocalized with RXR*α* (Figure 3(a)). We also found the same phenomenon in primary spinal neurons cultured *in vitro*; that is, the expression of p66shc in spinal cord neurons before injury was significantly lower than that after injury, and p66shc colocalized with RXR*α* in neuronal cells (Figure 3(b)). Based on the above research results, we speculate that RXR*α* may participate in the regulation of neurite regeneration after spinal cord injury by regulating p66shc expression.

### 3.4. RXR*α* Regulated p66shc Expression after Spinal Cord Injury

**Figure 4:**
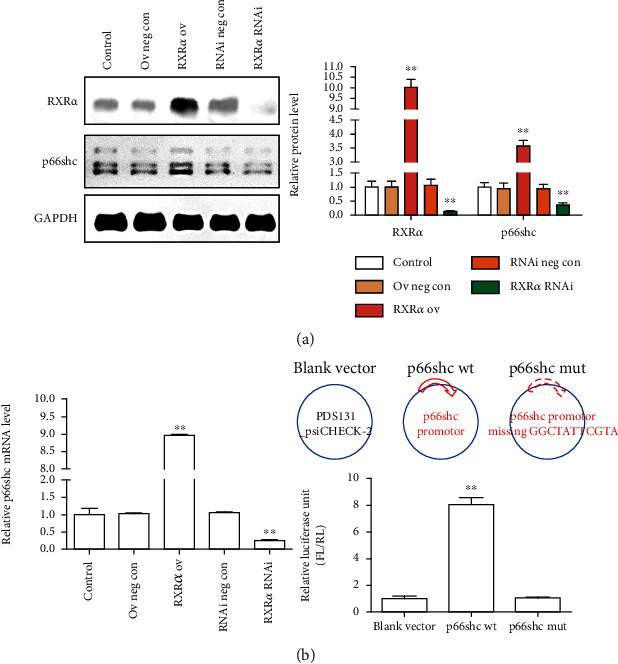
RXR*α* promoted p66shc expression after spinal cord injury. (a) Western blot analysis showed that RXR*α* overexpression promoted p66shc expression in primary spinal cord neurons cultured *in vitro*, while RXR*α* interference inhibited p66shc expression in primary spinal cord neurons cultured *in vitro*. (b) Real-time PCR and luciferase assays showed that RXR*α* inhibited the expression of p66shc at the mRNA level by binding to the promoter region of p66shc. *N* = 5/group; ^∗∗^vs. control, *P* < 0.01.

To further verify the regulatory relationship between RXR*α* and p66shc, we constructed RXR*α* overexpression and interference lentiviral plasmids to observe whether RXR*α* regulated p66shc expression in neuronal cells. The results showed that RXR*α* overexpression promoted p66shc expression in neurons, while RXR*α* interference also inhibited p66shc expression in neurons (Figure 4(a)). Therefore, RXR*α* regulates p66shc expression. Does RXR*α* play a direct or indirect role in the regulation of p66shc expression? We first examined the effect of RXR*α* overexpression or interference on the mRNA level of p66shc. We found that after RXR*α* overexpression, the expression of p66shc increased at the mRNA level; after RXR*α* interference, the expression of p66shc decreased at the mRNA level. Therefore, a luciferase assay was performed to further verify whether RXR*α* can bind to the promoter region of p66shc. The results showed that RXR*α* could indeed bind to the promoter region of p66shc and participate in the regulation of p66shc expression (Figure 4(b)).

### 3.5. RXR*α* Inhibited Neurite Regeneration after Spinal Cord Injury by Downregulating p66shc Expression

**Figure 5:**
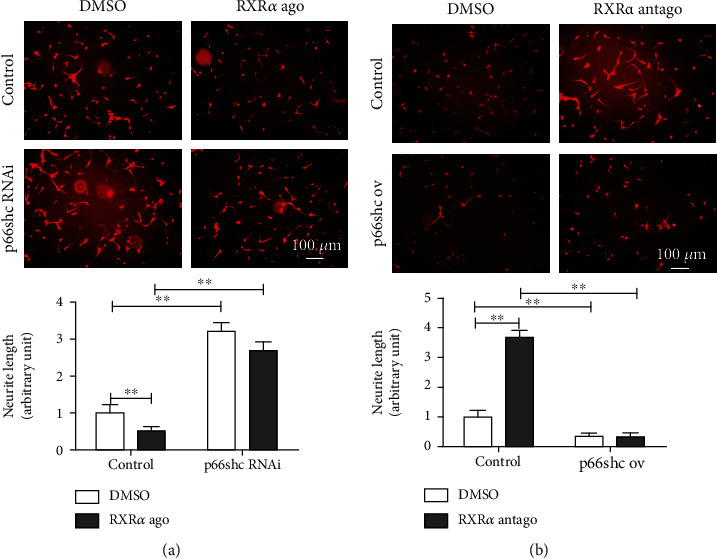
RXR*α* inhibited neurite regeneration after spinal cord injury by downregulating p66shc expression. (a) RXR*α* agonist significantly inhibited neurite regeneration of the cultured primary spinal cord neurons; this effect can be blocked by p66shc interference in the *in vitro* cultured primary spinal cord neurons. (b) RXR*α* antagonist significantly promoted neurite regeneration of the cultured primary spinal cord neurons; this effect was blocked by p66shc overexpression. *N* = 5/group. ^∗^vs. control, *P* < 0.05; ^∗∗^vs. control, *P* < 0.01; scale bar: 100 *μ*m.

Based on the above results, we further verified the effect of RXR*α* targeting of p66shc on neurite regeneration after spinal cord injury. We added an RXR*α* agonist to spinal cord neurons with p66shc interference and found that the RXR*α* agonist did not inhibit neurite regeneration after spinal cord injury. Similarly, we added an RXR*α* antagonist to spinal cord neurons overexpressing p66shc and found that the RXR*α* antagonist did not promote neurite regeneration after spinal cord injury (Figures 5(a) and 5(b)). The above results demonstrate that RXR*α* inhibited neurite regeneration after spinal cord injury by downregulating p66shc expression.

## 4. Discussion

The loss of body function caused by spinal cord injury severely affects the quality of life of patients. Promoting neurite regeneration and functional recovery after spinal cord injury is an urgent issue that needs to be addressed [1, 11]. Current treatment methods for spinal cord injury include high-dose hormone use at the early stage and rehabilitation at the late stage [12–14]. The study of nerve regeneration after spinal cord injury will provide a basis for the treatment of spinal cord injuries. RXR, as a member of the nuclear hormone receptor superfamily, mediates the cellular biological effects of various hormones and drugs [15, 16]. The increase in retinal dehydrogenase activity after spinal cord injury is the first direct evidence that retinoid acid (RA) substances participate in the physiological response to spinal cord injury. Injury leads to a significant increase in retinaldehyde dehydrogenase 2 (RALDH2) activity, reaching a peak 8-14 days after injury [17, 18]. In the uninjured rat spinal cord, RALDH2 is only present in the meninges, oligodendrocytes, pericytes, and NG2-positive glial cell populations around the injury site [18, 19]. After spinal cord injury, the expression of RARs only changes slightly, but the cell distribution changes significantly. In noninjured tissue, RAR*α*, RXR*α*, and RXR*β* are found in the cytoplasm of motor neurons and glial cells. In the neurons that survive injury, there is nuclear localization of RAR*α*, RXR*α*, and RXR*β* [3]. In the context of increased local RA synthesis, the observation of the translocation of RARs to the nucleus indicates that neurons, glial cells, and macrophages are the targets of RA signaling after spinal cord injury, which is consistent with spinal cord development data [20–23]. Studies have shown that the RA-RAR*β* pathway may have transcriptional inhibitory effects on axon regeneration and myelination signaling pathways [6]. RAR*β* can bind to RXR and then bind to the promoter region of downstream target proteins to participate in signal regulation [24]. However, the role and regulatory mechanism of RXR*α* in rat spinal cord injury remain unclear. Using a rat spinal cord injury model, we observed that RXR*α* played a role in inhibiting neuronal regeneration after spinal cord injury. This effect can be achieved by regulating the aging-related and oxidative stress-related protein p66shc.

Studies have shown that the activation of p66shc by redox stress is associated with a shortened life span and mitochondrial dysfunction. However, it is still unclear how p66shc provides neuroprotective effects on secondary stress. After an injury response or an oxidative stress response, p66shc relocates to the nucleus and mitochondria to participate in the regulation of mitochondrial function and oxidative stress [25, 26]. After spinal cord injury, severe oxidative stress responses occur in the injured area, and injured cells simultaneously initiate 2 signaling pathways: apoptosis and repair. In p66shc knockout mice, hippocampal neurons maintain certain nerve regeneration and participate in the regulation of hippocampal neuron aging [27, 28]. Is p66shc associated with nerve regeneration after spinal cord injury? Our study found that the expression of p66shc was upregulated after spinal cord injury. The upregulation of p66shc as an aging gene promoted cell senescence, and the regenerative ability of the injured neurons was reduced. The increased expression of the aging gene p66shc further inhibited neurite regeneration. Is the increase in p66shc expression regulated by RXR*α*? We used *in vivo* and *in vitro* studies to demonstrate that RXR*α* binds to the promoter region of p66shc and promotes the expression of p66shc, thereby further inhibiting regeneration after spinal cord injury. p66shc is a protein associated with the extensive regulation of cellular oxidative stress and aging. Its physiological and pathological functions are not limited to the inhibition of neurite regeneration after spinal cord injury. We speculate that p66shc is also involved in regulating the survival and aging of spinal cord neurons after injury. Neuronal survival and aging also play important roles in the repair of spinal cord injury, which needs to be further studied.

As a transcription factor, RXR*α* regulates the expression of a variety of target genes. As a senescence-related and oxidative stress-related protein, p66shc is also widely involved in the regulation of cell functions. This study only focused on RXR*α* and its downstream target gene p66shc, resulting in certain limitations. However, both RXR*α* and p66shc have wide ranges of functions in the body, and their roles in spinal cord injury are not clear. Therefore, we first need to clarify the function of RXR*α* and p66shc after spinal cord injury and then further study the mechanism of action. The present study demonstrates that RXR*α* and p66shc play negative regulatory roles in neural regeneration after spinal cord injury. RXR*α* achieves this negative regulatory function through the targeted promotion of p66shc expression. However, in the inhibition of regeneration after spinal cord injury, in addition to regulating the expression of p66shc, are more downstream target genes involved in the regulation of neuronal function after spinal cord injury? In addition to inhibiting neurite regeneration, does p66shc also inhibit repair after spinal cord injury by exacerbating oxidative stress and promoting neuronal apoptosis and aging? Recent research reported that repressor activator protein 1 (Rap1), an established telomere-associated protein, which is essential for the maintenance of telomere structural integrity, acted as a novel modulator of hypoxia-induced apoptosis in myocardial ischemia/reperfusion injury [29]. Telomere, which defines the ends of chromosome, has close relationship with cell senescence and apoptosis [30]; therefore, Rap1 is an important senescence-associated protein. p66shc is an oxidoreductase that promotes mammalian aging by producing reactive oxygen species in mitochondria, hydrogen peroxide (H_2_O_2_) stress was used as an artificial means of aging in the cells, and this resulted in RAP1 levels decreasing; whether Rap1 is related to neuronal apoptosis, aging, and regeneration after SCI remains to be further studied; we speculate that p66shc promotes the production of reactive oxygen species in mitochondria, decreases the level of telomere binding protein Rap1, and shortens telomere lengths, leading to cell senescence; in-depth research is still needed to address these questions.

## Data Availability

The accessibility data used to support the findings of this study were collected according to scientific research criteria and can be available from the corresponding authors upon request.
